# Principals’ reports of adults’ alcohol use in Australian secondary schools

**DOI:** 10.1186/s12889-016-2877-4

**Published:** 2016-02-29

**Authors:** Bernadette M. Ward, Rebecca Kippen, Penny Buykx, Geoffrey Munro, Nyanda McBride, John Wiggers

**Affiliations:** School of Rural Health, Monash University, PO Box 666, Bendigo, Victoria 3552 Australia; Melbourne School of Population and Global Health, University of Melbourne, Melbourne, Australia; School of Health and Related Research, University of Sheffield, Sheffield, UK; Australian Drug Foundation, Melbourne, Australia; National Drug Research Institute, Curtin University, Perth, Australia; School of Medicine and Public Health, University of Newcastle, Newcastle, Australia

**Keywords:** Alcohol, Schools, Principals, Adults

## Abstract

**Background:**

Schools provide opportunities for parents and the wider community to connect and support the physical and emotional wellbeing of their children. Schools therefore have the potential to play a role in the socialisation of alcohol use through school policies and practices regarding consumption of alcohol by adults at school events in the presence of children.

**Methods:**

This survey was undertaken to a) compare the extent to which alcohol is used at secondary school events, when children are present, in the states of New South Wales (NSW) and Victoria (VIC), Australia; b) describe principals’ level of agreement with these practices; c) their awareness of state policies on this issue; and d) the predictors of such events. A random sample of secondary schools, stratified to represent metropolitan and non-metropolitan schools were invited to participate. Bivariate and multivariate analysis were conducted with *p* values < 0.05 considered significant.

**Results:**

A total of 241 (43 %) schools consented to participate in the study. Fifteen percent of participating NSW schools and 57 % of VIC schools held at least one event in which alcohol was consumed by adults in the presence of children in the year before the survey. Of the 100 reported events, 78 % were Year 12 graduation dinners, and 18 % were debutante balls. Compared to NSW principals, VIC principals were significantly more likely to agree with the use of alcohol at these events; significantly less likely to be aware of their state education department policy on this issue; have a policy at their own school or support policy that prohibits alcohol use at such events; and less likely to report having enough information to make decisions about this.

**Conclusions:**

There is a growing focus on adults’ use of alcohol at school events when children are present. Schools can play an important role in educating and socialising children about alcohol via both the curriculum and policies regarding adults’ alcohol use at school events. Findings from this study suggest education department and school-based policies that prohibit or restrict the use of alcohol, are significant predictors of adults’ alcohol use at school events when children are present.

## Background

Widespread use of alcohol is associated with significant harm for young people [1]. In 2013, 26 % of Australians aged 14 years and older reported being a victim of an alcohol-related harm [2]. Schools, along with parents [3, 4], peers [5] and the broader community [6], can be influential in the socialisation of children to alcohol. Schools can provide opportunities for the intellectual, physical and emotional development of children with regard to their teaching, and role modelling regarding responsible alcohol consumption [7]. This ability to influence children is often focused on the curriculum and related programs [8] but schools also play a role in the socialisation of children to alcohol through school policies and practices that focus on consumption of alcohol by adults in the presence of children [9].

There have been several media reports of adults’ consuming alcohol at school events when students are present in Australia, England and the US [10–14], although little published research on this topic exists. In Australia, to varying degrees, attempts to manage such consumption are embedded within liquor licensing acts and state and territory government education department policies and guidelines [9]. In most jurisdictions, much of the responsibility for decision-making on this issue rests with principals and school councils [9]. For example, in the state of VIC, the government policy states that decision-making is at the school level [15], whereas in the state of New South Wales, (NSW) the government policy indicates that alcohol must not be consumed or brought to school premises or functions, including those conducted outside school premises at any time when school students are present [16].

Internationally, there is a lack of policies and guidelines on this issue. The Australian National Council on Drugs (ANCD) guidelines on alcohol fundraising in schools, recommends that children not be exposed to or involved in such activities [17]. Australian parents’ support for such a recommendation is illustrated by the findings of a survey of 479 parents, in which 60 % disagreed with adults purchasing and consuming alcohol at school events [18]. Qualitative research with 14 schools in the state of VIC, Australia suggests that implementation of the state’s policy regarding adults’ use of alcohol at government schools when students are present varies between schools. Ten of the principals reported adults’ use of alcohol at school events when students were present. Twelve of the principals expressed concern about adults using alcohol at school events, with seven making changes to alcohol policies in recent years to remove/reduce the availability of alcohol for adults’ consumption. This is consistent with the findings of a survey of 218 Australian secondary school principals who reported that adult drinkers were often undesirable role models for students and that there was a need to change the culture of drinking amongst some parents [19]. Despite such findings, the extent to which alcohol consumption by adults occurs in the presence of school children has not been systematically quantified, nor has the extent of principal agreement or disagreement with this practice been described. Given this evidence gap and the differences in policy approaches between jurisdictions, a study was undertaken to a) quantify and compare the extent to which alcohol is used at NSW and VIC secondary school events when students are present; b) describe principals’ level of agreement with these practices; c) their awareness, support of state policies and need for further information on this issue; and d) the predictors of schools holding such events.

## Method

### Study design, setting and sample

A cross-sectional study was undertaken in the states of NSW and VIC, Australia in November 2014.

In NSW and VIC there are 398 and 395 secondary schools respectively, that include a range of year levels from 7–12 (equivalent to 12–19 years old) [20, 21]. Schools were excluded from the study if they were located in remote areas [22] (13 in NSW and 6 in VIC) [23, 24] or had been invited to or participated in previous qualitative research by the same authors on this topic. The remaining 385 NSW and 329 VIC schools were stratified to be representative of counts of ‘metropolitan’ and ‘non-metropolitan’ postal areas within each state [22]. Eighty percent (75 % in NSW and 84 % in VIC) of these schools were randomly selected and invited to participate.

### Recruitment and data collection procedures

A pre-survey letter was posted to school principals to introduce, explain and solicit support for the study prior to telephone contact. Five trained interviewers made up to six telephone calls to each selected school to establish contact and up to five additional calls to gain agreement to participate in a computer-assisted telephone interview (CATI). For each school, the principal or their delegate was invited to participate in the quantitative survey. Where there was an answering machine response a scripted message was left. All calls were made between 9 am and 5.30 pm.

### Measures

In the absence of published quantitative literature regarding the study aims, the questionnaire was developed based on policy documents and previous qualitative research involving secondary school principals [9, 12] (Ward B, Buykx P, Munro G, Wiggers J. Are schools and alcohol a good mix? Adults’ use of alcohol in Australian Secondary Schools (submitted and under review). BMJ Open). Skip functions were included to accommodate questions on a range of different events and school year levels. The survey was pre-tested by the researchers and piloted with nine principals in NSW and VIC to assess face and content validity. In addition to sociodemographic data about the school and characteristics of the principal, the questionnaire addressed the following domains:

- Consumption of alcohol by adults at school events

Participants were asked to report on the consumption of alcohol by adults at eight different types of school events, at which students were present, in the last 12 months (or in the number of months they had worked at the school if less than 12) (yes/no/don’t know). Where the response was ‘yes’, participants were asked if the alcohol was ‘bring your own’ (BYO) and/or sold and/or freely given (yes/no). For each of the possible events, participants were asked if they supported adults being able to consume at an event (agreement - scale of 1 to 5).

- Alcohol offered in a raffle or part of a prize

Principals were asked if, in the past 12 months (or months if less than a year) had alcohol (e.g. bottled wine or beer) been offered as a prize or part of a prize for school fundraising (yes/no/don’t know). Principals were then asked if they supported adults being able to purchase and consume alcohol in the presence of students at fundraising events (agreement - scale of 1 to 5).

- Policy and guidelines

Principals were asked about their awareness of state education department policy or guidelines regarding alcohol consumption at school functions at which students are present, whether their school had a policy or guidelines in place regarding such alcohol use, and if yes, whether it prohibited alcohol consumption, and whether the consumption of alcohol was permitted with school council or principal approval; and finally, whether they believed that the state education department should have a policy that prohibited the consumption of alcohol at all school functions at which students are present (yes/no/don’t know).

- Adequacy of, and need for information and resources

Principals were asked, using a scale of 1 to 5, whether they had adequate information to make decisions about the consumptions of alcohol at school functions. A sub-sample of 201 participants were asked if they, the school council or board would like additional information and/or skills to help make decisions about adults’ use of alcohol at school events. The principals were also asked the likelihood that they would use additional information if made available by the following means: face-to-face, online, by phone/ipad/other app, telephone support or mail-out information (five point Likert scale).

### Analysis

Statistical analyses were conducted using SPSS Statistics v.21. Chi-square tests were used to investigate differences in survey responses between NSW and VIC principals (Tables 1, 2, 3 and 4). We used logistic regression (Table 5) to determine whether alcohol consumption at any school events by adults in the presence of children was predicted by school location and year levels, principals’ dis/agreement, alcohol-policy awareness, support and need for further information on this issue. For all analyses, a *p*-value of less than 0.05 was considered statistically significant.

**Table 1:** Please indicate whether, to your knowledge, alcohol has been consumed by adults at any of the following functions organised by your school, either on or off school premises

Number of schools with response ‘yes’	NSW	Vic	Total
1 type of event			
School fete or fair	0	0	0
Sports day or other school sporting events	0	0	0
Year 10 graduation or valedictory dinner	0	2	2
Year 12 graduation or valedictory dinner	17	48	65
Debutante ball	0	5	5
Year 7/8 camp	1	0	1
Year 9/10 camp	0	0	0
Year 11/12 camp	0	0	0
2 types of events			
Graduation or valedictory dinner year 12; Debutante ball	1	11	12
3 types of events			
School fete or fair; Graduation or valedictory dinner year 12; Debutante ball	0	1	1
Total events	20	80	100
Schools to hold at least one event	19	67	86
Total schools	124	117	241
*% schools to hold at least one event* ^a^	*15.3*	*57.3*	*35.7*

^a^Differences between states significant at the 0.1 % level (chi-square)

**Table 2:** Principals’ level of agreement with adults’ use of alcohol at school events (when children are present) by state (%)

	n^a^		% Strongly agree/agree		% Neither agree/nor disagree		% Strongly disagree/disagree	
	NSW	Vic	NSW	Vic	NSW	Vic	NSW	Vic
School fete or fair^b^	121	110	0.0	1.8	1.7	9.1	98.3	89.1
Sports day or other school sporting events	121	110	0.0	0.0	0.8	0.0	99.2	100.0
Year 10 graduation or valedictory dinner	114	103	0.0	1.9	0.0	1.0	100.0	97.1
Year 12 graduation or valedictory dinner^c^	118	101	7.6	48.5	4.2	12.9	88.1	38.6
Debutante ball^b^	118	108	2.5	11.1	8.5	9.3	89.0	79.6
Year 7/8 camp	115	105	0.9	0.0	1.7	0.0	97.4	100.0
Year 9/10 camp	115	109	0.9	0.0	1.7	0.0	97.4	100.0
Year 11/12 camp	118	101	0.8	0.0	2.5	0.0	96.6	100.0
Fundraising events	124	116	3.0	10.3	6.5	11.2	89.5	78.4

^a^Not all principals responded to these questions

^b^Differences between states significant at the 5 % level (chi-square)

^c^Differences between states significant at the 0.1 % level (chi-square)

**Table 3:** Principals’ awareness and use of policy/guidelines regarding adults’ consumption of alcohol at school functions at which students are present (*n* = 241, NSW = 124, Vic = 117)

In regard to adults’ consumption of alcohol at school functions at which students are present:	n		% Yes		% No		% Don’t know/refused	
	NSW	Vic	NSW	Vic	NSW	Vic	NSW	Vic
As far as you are aware does your Education Department have a policy or guidelines?^d^	124	117	96.8	76.1	0.8	8.5	2.4	15.4
Does your school have a policy or guidelines?^c^	124	117	67.7	47.0	29.8	47.0	2.4	6.0
Does your school policy prohibit at all school functions?(a)^d^	84	55	96.4	63.6	2.4	34.5	1.2	1.8
Does your school policy allow with discretion of the principal? (a)(b)	84	55	19.0	29.1	79.8	65.5	1.2	5.5
Does your school policy allow with school council approval? (a)(b)^d^	84	55	9.5	56.4	81.0	34.5	9.5	9.1
Do you believe that your Education Department should have a policy/guidelines that prohibits alcohol at all school functions?^d^	124	117	91.9	53.8	6.5	41.9	1.6	4.3

(a) *N* = 139 Only applies to schools that have a policy

(b) In Victoria, the DEECD policy gives the principal/school council discretion

^c^Differences between states significant at the 1 % level (chi-square)

^d^Differences between states significant at the 0.1 % level (chi-square)

**Table 4:** Principals’ views on adequacy of information to make decisions about alcohol consumption at school functions, whether they would like additional information, and likely use of selected modalities

	NSW	Vic	NSW	Vic	NSW	Vic	NSW	Vic
Do you feel you have adequate information to make decisions about the consumption of alcohol at school functions?^c^	n		% Yes, completely adequate information		% Yes, somewhat adequate		% Not sure/No, somewhat inadequate	
	124	117	80.6	50.4	17.7	42.7	1.6	6.8
Would you, your school council or board like additional information and/or skills to help you make decisions about adults’ use of alcohol at school events?^b^	n		% Yes		% No		% Don’t know	
	101	100	23.8	44.0	76.2	54.0	0.0	2.0
Likely use:	n		% Likely/very likely		% Unsure		% Unlikely/very unlikely	
Online information package^a^	101	100	57.4	75.0	8.9	6.0	33.7	19.0
Telephone information--you call	101	100	44.6	57.0	7.9	6.0	47.5	37.0
Mailout information package	101	100	44.6	54.0	5.9	10.0	49.5	36.0
Phone/ipad/other ‘app’	101	99	33.7	40.4	6.9	14.1	59.4	45.5
Face-to-face session^b^	101	100	10.9	26.0	6.9	12.0	82.2	62.0
Telephone information--calls you	101	100	12.9	23.0	5.9	9.0	81.2	68.0

^a^Differences between states significant at the 5 % level (chi-square)

^b^Differences between states significant at the 1 % level (chi-square)

^c^Differences between states significant at the 0.1 % level (chi-square)

**Table 5:** Logistic regression analysis. Dependent variable is whether a school held at least one event in the previous year (fete/fair; sports day; Year 10 or 12 graduation dinner; debutante ball; Year 7/8, 9/10, 11/12 camp) at which alcohol was consumed by adults (yes = 86; no = 155; *n* = 241)

	n	Odds ratio	95 % confidence interval
State			
Reference category: New South Wales	124	1.00	
Victoria	117	3.26^b^	(1.52–7.02)
As far as you are aware, does your State Education Department have a policy or guidelines in place regarding alcohol consumption at school functions at which students are present?			
Reference category: Yes	209	1.00	
No	11	3.07	(0.68–13.78)
Don’t know	21	0.59	(0.18–1.94)
Does your school have a policy or guidelines in place regarding alcohol use at school functions at which students are present?			
Reference category: Yes, prohibits at all school functions	116	1.00	
Yes, does not prohibit at all school functions	21	26.38^b^	(3.01–231.47)
No policy	92	1.79	(0.84–3.82)
Don’t know/refused	12	0.80	(0.18–3.60)
Do you believe that your State Education Department should have a policy that prohibits the consumption of alcohol at all school functions at which students are present?			
Reference category: Yes	177	1.00	
No	57	3.69^b^	(1.60–8.51)
Don’t know	7	2.82	(0.45–17.67)
Do you feel you have adequate information to make decisions about the consumption of alcohol at school functions?			
Reference category: Yes, completely adequate information	159	1.00	
Yes, somewhat adequate	72	2.52^a^	(1.18–5.41)
Not sure/No, somewhat inadequate	10	4.02	(0.68–23.66)
School has Year 12^c^			
Reference category: No	15	1.00	
Yes	226	5.52^a^	(1.16–26.30)

^a^Differences significant at the 5 % level

^b^Differences significant at the 1 % level

^c^This analysis controls for whether the sampled schools have Year 12, since most reported events were Year 12 graduation or valedictory dinners

### Ethics approval

Approval was granted by the NSW Department of Education &Communities, the VIC Department of Education &Training, Australian Drug Foundation, Curtin, Newcastle and Monash Universities.

## Results

### School sample and characteristics

Of the 567 (NSW = 290, VIC = 277) randomly sampled schools, 124 (43 %) NSW and 117 (42 %) VIC schools consented to participate providing a total sample of 241 (43 %) schools.

Of the 241 participating schools, 124 (51 %) were in NSW and 117 (49 %) in VIC.

Forty-four percent of all schools and within each state (Sydney = 54, Melbourne = 51) were in metropolitan postal areas and 56 % (rest of NSW = 70, rest of VIC = 66) were in non-metropolitan postal areas. The majority (94 %) of participating schools involved school years 7–12 (students aged approximately 12 to 19 years).

#### Participants

Eighty-one percent of participants were principals, 15 % were deputy/assistant principals and 4 % were senior teachers (hereafter referred to as ‘principals’). Of these 61 % was male and 67 % were aged 50 years or more. There were no significant differences in these characteristics between states. There was a significant difference in mean length of employment at the school for NSW (*M =* 6.3, *SD =* 5.3) and VIC (*M =* 10.4, *SD =* 8.7); *p* < 0.001 principals (range 1–38 years).

### Alcohol at events when students are present

Thirty-six per cent of principals (NSW = 15 %, VIC = 57 %) reported at least one event where alcohol was consumed in the presence of students in the previous 12 months (see Table 1), with significant differences between the two states (*p* = < 0.01). One school (VIC) held three events, 12 schools (NSW = 1, VIC = 11) each held two events and 73 (NSW = 18, VIC = 55) schools held one event. Of the 100 reported events, the majority were Year 12 graduation/valedictory dinners (78 %) and debutante balls (18 %), with two Year 10 graduation/valedictory dinners, one school fete or fair and one Year 7/8 camp constituting the remainder. No alcohol consumption was reported at sporting events, Year 9/10 or 11/12 camps. There were 42 reports of alcohol being offered as a prize for a school fundraiser with 25 of these in NSW and 17 in VIC but the difference between states was not significantly different (not shown).

### Principals’ level of agreement with adults consuming alcohol at school events when students are present

As shown in Table 2, the majority of both NSW and VIC principals disagreed or strongly disagreed with the consumption of alcohol occurring at school sports events, Year 10 graduation dinners and school camps. NSW principals were more likely than their VIC counterparts to disagree with the consumption of alcohol at school fetes (98 % vs 89 %), Year 12 graduation dinners (88 % vs 39 %), debutante balls (89 % vs 80 %) and school fundraisers (90 % vs 78 %), with differences between states being statistically significant for the first three of these.

### Awareness of policies/guidelines

As per Table 3, NSW principals were significantly more likely than their VIC counterparts to be aware of their state education department policy (97 % vs 76 %), have a policy at their own school (68 % vs 48 %), for that policy to prohibit adults’ consumption of alcohol when students are present (96 % vs 64 %) and to support education department policy that prohibits such use (92 % vs 54 %).

#### Information and resources

Overall, 159 (66 %) of principals reported they have completely adequate information to make decisions about alcohol consumption at school functions and this was significantly more likely for NSW principals (81 %) than VIC participants (50 %). Of the 201 participants that were asked about information preferences to help support decision-making regarding adults’ use of alcohol at school events, 34 % indicated that they would like additional information (NSW = 24 %, VIC = 44 %) and the difference between states was statistically significant (*p* <0.01). Of the suggested modalities of information provision, there was a clear preference for online information. The preference for both online and face-to-face information was significantly different between states, with VIC principals reporting they were more likely to use these resources than their NSW counterpart (see Table 4).

#### Predictors of adults’ alcohol use at school events

Logistic regression analysis (see Table 5) shows that, controlling for other variables, schools were significantly more likely to have held at least one event in the previous year where alcohol was consumed by adults if they were located in VIC (compared to NSW); included Year 12 level; the school did not have a policy prohibiting the use of alcohol use at school functions at which students were present; the principal did not believe that their state education department should have a policy that prohibits alcohol consumption at such events; and the principal felt that s/he had ‘somewhat’ adequate information to make decisions about this issue (compared to those who felt they had ‘completely adequate’ information).

## Discussion

This study sought to quantify and compare to extent to which alcohol is used at NSW and VIC secondary school events when students are present; principals’ awareness and level of agreement with these practices and the predictors of schools holding such events. The study found that fifteen percent of participating NSW schools and 57 % of VIC schools held at least one such event in the year before the survey. The majority of principals disagreed/strongly disagreed with the practice and NSW principals were significantly more likely to disagree than their VIC counterparts. NSW principals were also significantly more likely to be aware of, support policies and have adequate information to make decisions about alcohol at such events. Significant predictors of alcohol at these events were location (VIC), year level, absence of school policy and support for state education policy that prohibited adults’ alcohol use at these events and inadequate information to make a decision about the issue.

The significant differences between the prevalence of and state principals’ awareness of and support for adults’ consumption of alcohol at school events when children are present may be explained by underlying alcohol related differences between NSW and VIC. While there are no significant differences in the patterns of drinking between the two populations [2], the NSW population is more likely to support restrictive alcohol policies [25]. Consistent with this, the VIC state education department policy that leaves much of the decision-making on this issue to the principal. In contrast, the NSW policy is more explicit, prohibiting the consumption of alcohol at any school event when children are present. More specifically, in VIC, there are different education department guidelines for functions that are held on or off school premises. When VIC school functions are held at off-site at licensed premises, students may be present under the supervision of a parent/guardian [15] and VIC principals report the responsibility for meeting liquor licensing requirements does not sit with the school. In contrast, the education department policy in NSW does not differentiate between functions on or off school premises [16]. Nevertheless, where there is a teacher-student relationship at a school hosted event, irrespective of parental attendance or location, school staff have a duty of care to ensure that students are safe [26, 27]; irrespective of adults’ drinking behaviours.

The explicit NSW policy on adults’ use of alcohol in schools when children are present may influence principals’ attitudes and beliefs about other uses of alcohol in schools. The use of alcohol in school fundraising is not explicit in the NSW policy but may be implied. In VIC, the education department policy is explicit that “students should not be involved in fundraising events which have an alcoholic beverage as a prize” [15]. Despite this, 3 % of NSW and 10 % of VIC principals reported they agree/strongly agree with alcohol being used for fundraising (when children are present) while a further 6 and 11 % respectively neither agree nor disagree. The relative autonomy that VIC principals have on the overall use of adults’ use of alcohol at school events, may be a stronger predictor of their use of alcohol for fundraising purposes than an explicit state policy or national guidelines on the use of alcohol for this purpose [17].

Adults’ alcohol use at school events when children are present was significantly associated with principals’ reports of information they had to make decisions about this issue. School principals may be seeking information to assist them in their decision-making about the pros and cons of exposing students to alcohol at school events. The existing Australian state and territory policies about adults’ use of alcohol at school events are conflicting [9]. The development of national guidelines, similar to those on the use of alcohol for fundraising in schools [17], could inform state and territory policies and principals seeking information and guidance on this issue.

There is limited evidence on the prevalence of adults’ alcohol use in schools and the potential long-term influence of this on children. The majority of events reported in this study were Year 12 graduations and this may reflect the legal age of purchase of alcohol in Australia (18 years) and the normalisation of alcohol use at social events. However, many school events include other family members; often younger children. Evidence from meta-analyses suggests children form memory associations of alcohol before they ever drink alcohol themselves [28] and that these memories are associated the frequency of parental drinking and adolescents’ subsequent initiation to and misuse of alcohol [29]. Adults’ use of alcohol at school events, in the presence of children, may be infrequent particularly when compared to the use of alcohol in other public and/or private settings. However the negative role modelling of alcohol consumption reported at some school events [12] may be exacerbating the significant alcohol-related harm experienced by young people [1]. Schools may be underestimating their role in socialising children to alcohol use.

In some countries there have been efforts to reduce the consumption of alcohol at school events’ particularly when children are present. In England, there have been calls to prohibit the sale of alcohol in schools [11] and recent legislative changes in New Zealand have further restricted the use of alcohol at school events [30]. In contrast, in some Australian jurisdictions the licensing requirements to provide alcohol at school events when children are present have been reduced [31, 32] to the extent that, within limits, schools in these jurisdictions (including NSW) no longer need to apply for a liquor licence to sell or supply alcohol.

The reports of adults’ alcohol use at schools in this study may, compared to the use of alcohol in broader society, appear to be relatively benign. However, it is likely there is under-reporting of adult drinking at secondary school functions when children are present because of a social desirability bias by those who did/not participate. Unsurprisingly, a large number of schools that were contacted chose not to participate. While this is typical of school-based surveys [33], it may also reflect a reluctance to report adult drinking at secondary school functions when children are present. Our survey was limited to government secondary schools in two states. Qualitative reports from VIC secondary school principals suggest that adults’ alcohol use at Catholic and primary school functions may be higher than in secondary schools. Primary schools are more likely to host events where adults *and* children are involved (e.g. fetes, school concerts) where adults may regularly use alcohol in the presence of young children. Further research is needed to examine the extent of alcohol use at primary schools and the extent to which safeguards are in place to protect the wellbeing of children and other community members. Efforts to examine and/or further restrict adults’ use of alcohol at school events when students are present are only one component of a broader approach to a more responsible societal attitude to alcohol.

## Conclusions

Findings from this study suggest education department and school-based policies that prohibit or restrict the use of alcohol, are significant predictors of adults’ alcohol use at school events when children are present. These events are significantly more likely to be hosted by schools where the principals agree with such practices and have significantly lower levels of awareness of education department policies on this issue. However, these principals are also significantly more likely to report that they need further information on which to base such decisions. Education departments have an important role in ensuring alcohol policy is responsive to the needs of principals charged with ensuring the wellbeing of children and young people.

## Abbreviations

ANCD: Australian National Council on Drugs
BYO: Bring your own
CATI: Computer assisted telephone interview
NSW: New South Wales
VIC: Victoria
